# Zinc/Magnesium Ferrite Nanoparticles Functionalized with Silver for Optimized Photocatalytic Removal of Malachite Green

**DOI:** 10.3390/ma17133158

**Published:** 2024-06-27

**Authors:** Ricardo J. C. Fernandes, Beatriz D. Cardoso, Ana Rita O. Rodrigues, Ana Pires, André M. Pereira, João P. Araújo, Luciana Pereira, Paulo J. G. Coutinho

**Affiliations:** 1 Physics Centre of Minho and Porto Universities (CF-UM-UP), University of Minho, Campus de Gualtar, 4710-057 Braga, Portugal; 2 LaPMET—Associate Laboratory, 4169-007 Porto, Portugal; 3 CEB—Centre of Biological Engineering, University of Minho, Campus de Gualtar, 4710-057 Braga, Portugal; 4 LABBELS—Associate Laboratory, 4710-057 Braga, Portugal; 5 IFIMUP—Materials Physics Institute, University of Porto, R. Campo Alegre, 4169-007 Porto, Portugal

**Keywords:** magnetic nanoparticles, mixed ferrites, photocatalysis, photodeposited silver, visible light

## Abstract

Water pollution is a major environmental challenge. Due to the inefficiency of conventional wastewater treatment plants in degrading many organic complex compounds, these recalcitrant pollutants end up in rivers, lakes, oceans and other bodies of water, affecting the environment and human health. Semiconductor photocatalysis is considered an efficient complement to conventional methods, and the use of various nanomaterials for this purpose has been widely explored, with a particular focus on improving their activity under visible light. This work focuses on developing magnetic and photoactive zinc/magnesium mixed ferrites (Zn_0.5_Mg_0.5_Fe_2_O_4_) by sol-gel and solvothermal synthesis methods, which are two of the most important and efficient methods used for the synthesis of ferrite nanoparticles. The nanoparticles (NPs) synthesized by the sol-gel method exhibited an average size of 14.7 nm, while those synthesized by the solvothermal method had an average size of 17.4 nm. Both types possessed a predominantly cubic structure and demonstrated superparamagnetic behavior, reaching a magnetization saturation value of 60.2 emu g^−1^. Due to the high recombination rate of electrons/holes, which is an intrinsic feature of ferrites, surface functionalization with silver was carried out to enhance charge separation. The results demonstrated a strong influence of adsorption and of the deposition of silver. Several optimization steps were performed during synthesis, allowing us to create efficient catalysts, as proved by the almost full removal of the dye malachite green attaining 95.0% (at a rate constant of 0.091 min^−1^) and 87.6% (at a rate constant of 0.017 min^−1^) using NPs obtained by the sol-gel and solvothermal methods, respectively. Adsorption in the dark accounted for 89.2% of the dye removal for nanoparticles prepared by sol-gel and 82.8% for the ones obtained by the solvothermal method. These results make mixed zinc/magnesium ferrites highly promising for potential industrial application in effluent photoremediation using visible light.

## 1. Introduction

Water scarcity is considered one of the major problems affecting humanity [1]. Recent estimates show that by 2030, about one-third of the global population will face problems accessing drinking water [2]. Consequently, with the noticeable repercussions of global warming in the last decades, wastewater management has become one of the main topics of interest to prevent irreversible consequences in the future [3]. One of the main contributors to the decrease in the global quality of water systems is anthropogenic pollution [4]. Several pollutants, such as dyes, pesticides, personal care products, pharmaceuticals and per- and poly-fluoroalkyl substances (PFASs), among others, are increasingly present in water resources at concentrations ranging from a few ng/L to mg/L [5,6,7,8]. Furthermore, they can destroy ecosystems, affecting entire food chains. Concerning public health, most of these pollutants have been associated with disorders like immunotoxicity and reproductive diseases, with some considered carcinogenic [9]. Effluents from either industrial or domestic sources are usually fed to wastewater treatment plants (WWTPs), which are ineffective for the removal of more complex pollutants most of the time; thus, the development of new processes and technologies that can complement the existing ones is essential [10].

Semiconductor photocatalysis has been tested for the degradation of various pollutants [11,12]. This process is based on the absorption of light by a semiconductor, with energy similar to or higher than the band gap, forming reactive species by electron/hole migration to the semiconductor’s surface. Comparing with conventional biological processes, photocatalysis has the advantage of being non-selective, so it is efficient to eliminate a broad range of contaminants and break down complex pollutants into simpler substances like water, carbon dioxide and inorganic ions, and the photocatalysts can be reused multiple times [13]. This also paves the way for exploring the complementarity between both processes, potentially leading to the development of synergistic treatments [14]. However, concerns have been raised regarding the use of nanomaterials in the photocatalytic process due to their possible toxicity [14,15]. The use of photocatalytic and superparamagnetic nanoparticles (SMNPs) is one efficient strategy, as SMNPs can be retained after the process, preventing leaching in the final process.

Nanomaterials have garnered increased interest during the last decades due to their unique physicochemical stability, high reactivity and high surface area, which are essential for efficient interaction with pollutants [16,17]. Nanomaterials like TiO_2_ [18,19], iron oxides [20,21] and Ga_2_O_3_ [22,23] have shown good efficiency as photocatalysts towards various pollutants [24,25]. However, most of them require UV light, which can be considered limiting from an industrial perspective due to the high prices of artificial UV light. So, the use of visible light is becoming highly promising due to its high solar availability (45–50%) at the earth’s surface [26,27], but the lower availability of UV light in the solar spectrum (5%) is a handicap for the efficient use of direct sunlight as an energy source, and strategies to improve this use are needed. In processes that use NPs, their possible toxicity must be considered. However, by applying magnetic NPs, this problem is minimized or excluded since the NPs can be retained in the reactor and easily removed after the process by applying a magnetic field, so they are not released into the treated water.

Ferrite nanoparticles are widely used nanomaterials in technological applications, such as biosensors, permanent magnets, drug delivery systems, hyperthermia and photocatalysis. Their widespread use is attributed to their excellent physicochemical properties, including outstanding chemothermal stability, superior electro-optical characteristics and superparamagnetic properties [28]. In this last context, ferrite NPs have shown promising activity under visible light [29]. Their superparamagnetic behavior, narrow bandgap and enhanced biocompatibility make them exciting nanomaterials to explore [30]. Furthermore, ferrites can incorporate different constituents, which makes them very versatile NPs [31,32], and they have revealed promising results in degrading various pollutants, including dyes [33]. The combination of different constituents to develop mixed ferrites, such as Zn_0.5_Ca_0.5_Fe_2_O_4_ and Co_0.5_Ni_0.5_Fe_2_O_4_, can improve photocatalytic activity and other features of the NPs, such as enhanced magnetic behavior or affinity towards certain compounds [29,33]. Nonetheless, most ferrites need further functionalization due to the high recombination of electrons/holes, hampering their photocatalytic activity. The use of deposited silver has shown promising results in previous photocatalytic studies [34,35], as Ag can improve the charge separation, acting as an electron acceptor and promoting the formation of reactive species [25,26], considerably decreasing the high recombination rate in ferrites.

This work represents the first exploration of the photocatalytic activity of Zn_0.5_Mg_0.5_Fe_2_O_4_ NPs functionalized with silver. The core NPs were synthesized either via sol-gel or using the solvothermal method, and two time periods of Ag incorporation were studied (12 h and 24 h). To assess the photocatalytic activity of these NPs, the dye malachite green (MG) was used as model pollutant. MG dye, a widely used triphenylmethane dye in the silk dyeing, aquaculture and textile industries, is a common environmental pollutant posing significant threats to human health and ecosystems [36]. MG is toxic to all organisms and has been linked to carcinogenesis, mutagenesis, chromosomal fractures, teratogenicity and respiratory toxicity [36]. Previous results on MG removal by heterogeneous catalysis showed that different nanostructures based on ZnO and TiO_2_ can be efficient [37,38]. However, the synergy between adsorption and photocatalysis is underdeveloped, as well as the synthesis of superparamagnetic nanosystems capable of removing pollutants. This research evaluated the efficiency of these catalysts under dark and visible conditions and compared the two synthesis methods, the effect of silver photodeposition time and the importance of NP cleaning after synthesis by the solvothermal method and prior to silver photodeposition, as well as the effect of photocatalyst concentration on catalytic efficiency. This specific solvothermal method [39] has never been previously used to synthesize NPs for photocatalysis. This work provides novel insights into optimizing photocatalytic materials for enhanced environmental remediation, highlighting the significance of synthesis methods and functionalization in achieving superior photocatalytic performance.

## 2. Materials and Methods

### 2.1. Chemicals

The chemicals for NPs synthesis, i.e., iron(III) nitrate nonahydrate (≥98%), zinc(II) chloride dihydrate (≥98%), magnesium chloride (anhydrous, ≥98%), iron citrate tribasic monohydrate (18–20% Fe basis), silver nitrate (≥99%), citric acid (99%), nitric acid (ACS reagent, 70%), 1-octadecene (≥99.0% GC), oleic acid (≥99%), absolute ethanol (for spectroscopy Uvasol^®^, Darmstadt, Germany), methanol (for spectroscopy Uvasol^®^), dimethyl sulfoxide (DMSO, for spectroscopy Uvasol^®^), tetrahydrofuran (THF, for spectroscopy Uvasol^®^) and malachite green oxalate salt (for microscopy), were purchased from Sigma-Aldrich (St. Louis, MO, USA).

### 2.2. Synthesis of Zn_0.5_Mg_0.5_Fe_2_O_4_ Ferrites by Sol-Gel and Solvothermal Methods

Mixed zinc/magnesium ferrite NPs (Zn_0.5_Mg_0.5_Fe_2_O_4_) were synthesized by two different methods, sol-gel and solvothermal. In the sol-gel method, 2 mmol of iron(III) nitrate nonahydrate, 0.5 mmol of zinc(II) chloride and 0.5 mmol of magnesium chloride were well dispersed in water, with a 20 mL final volume [29]. Then 630.42 mg of citric acid was added to the well-dispersed solution, followed by 70 µL of nitric acid, with the solution in constant stirring. The mixture was then slowly heated until 90 °C, forming a gel, which was subsequently heated until a loose powder was obtained.

**Figure 1:**
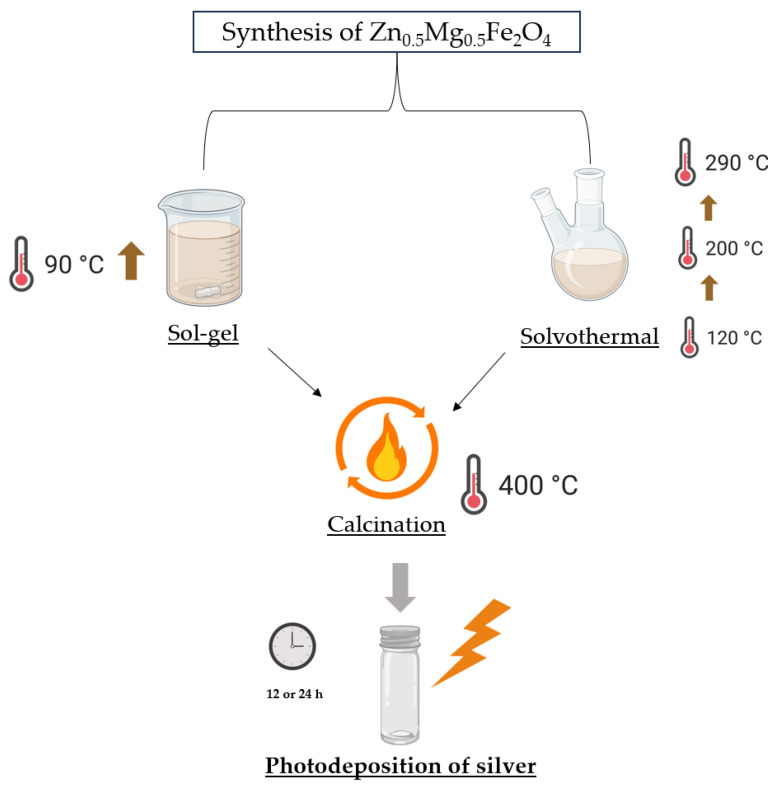
Schematic representation of the different steps of synthesis of NPsA@Ag and NPsB@Ag, from core Zn_0.5_Mg_0.5_Fe_2_O_4_ synthesis to functionalization with silver.

The preparation of Zn_0.5_Mg_0.5_Fe_2_O_4_ by the solvothermal route was based on the work of Cardoso et al. [39], but with some modifications. The reagents used were 0.5 mmol of magnesium acetate tetrahydrate, 0.5 mmol of zinc(II) chloride dihydrate, 2 mmol of iron citrate tribasic monohydrate and 3 mmol of oleic acid. Specifically, 15 mL of octadecene was placed in a double-neck flask and preheated to 120 °C. Next, the reagents were placed in the preheated solvent and left at 120 °C for 60 min. After this time, the reflux condenser was connected to the flask neck, and the solution was heated to 200 °C at a heating rate of 5 °C min^−1^ and maintained under these conditions for 100 min. The solution was then further heated at a heating rate of 1 °C min^−1^ until it reached 290 °C, and the reaction was left under reflux for an additional 60 min. The nanoparticles were washed with ethanol several times to eliminate the excess of oleic acid from the as-formed nanoparticles. At last, the nanoparticles were calcined at 400 °C for 30 min. After calcination, the nanoparticles were cleaned with THF and DMSO. Further cleaning was also performed using water and ethanol for several cycles. Figure 1 shows a schematic representation of the preparation of silver-functionalized nanoparticles.

### 2.3. Functionalization of Synthesized NPs with Silver

The synthesized nanoparticles, obtained by both the solvothermal and the sol-gel methods, were functionalized with silver following a previously reported method [29]. First, 30 mg of nanoparticles was well dispersed in 10 mL of ultrapure water. Next, 1.5 mL of a solution containing 1 mol L^−1^ of silver nitrate was slowly added to the nanoparticle solution and kept under constant agitation. A total of 1 mL of methanol was also added to the solution. Then UV light was continuously applied for 12 or 24 h using a 100 W Xenon arc lamp (LOT Oriel GmbH Co., Ltd., Darmstadt, Germany). The resulting solution was washed several times with ultrapure water and ethanol and kept in a drying oven at 80 °C overnight.

**Figure 2:**
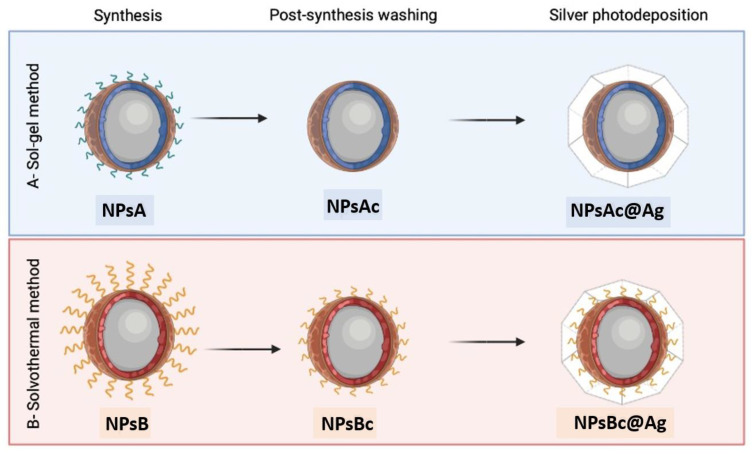
Schematic representation of the different nanoparticles synthesis routes.

Figure 2 shows a schematic representation of the prepared nanoparticles. NPsA and NPsB represent Zn_0.5_Mg_0.5_Fe_2_O_4_ nanoparticles prepared by the sol-gel (A) and solvothermal (B) methods, respectively. NPsAc and NPsBc are the same nanoparticles, but with a cleaning step after calcination. NPsAc@Ag and NPsBc@Ag represent the corresponding nanoparticles (NPsAc and NPsBc) functionalized with silver.

### 2.4. Structural Characterization of Synthesized NPs

Absorption spectra of Zn_0.5_Mg_0.5_Fe_2_O_4_ and Ag-functionalized Zn_0.5_Mg_0.5_Fe_2_O_4_ dispersions were measured in a double-beam Shimadzu UV/Vis/NIR spectrophotometer, model UV-3600 Plus (Shimadzu Corporation, Kyoto, Japan). X-ray diffraction (XRD) measurements were performed with a PAN’Alytical X’Pert PRO diffractometer (Malvern Panalytical Ltd., Malvern, UK) in a Bragg–Brentano configuration, operating with Cu K_α_ radiation (λ = 0.154060 nm) at the Electron Microscopy Unit of the University of Trás-os-Montes and Alto Douro (UTAD), Vila Real, Portugal. For the assessment of zeta(ζ)-potential, dynamic light scattering (DLS) equipment, Litesizer^TM^ 500 from Anton-Paar (Anton-Paar GmbH, Graz, Austria), equipped with a laser diode of λ = 658 nm was used. Sodium phosphate solution (0.1 mol L^−1^) and boric acid (0.2 mol L^−1^)/citric acid (0.05 mol L^−1^) mixed solution were used in different proportions to obtain buffers with several pH values. The assessment of magnetic properties was performed in a MPMS3 Superconducting Quantum Interference Device (SQUID) Quantum Design MPMS5XL magnetometer (Quantum Design Inc., San Diego, CA, USA) at IFIMUP (University of Porto, Portugal). Microscopy images of Zn_0.5_Mg_0.5_Fe_2_O_4_ NPs were obtained in a high-contrast transmission electron microscope (TEM), JEOL JEM-1010, operating at 100 kV (Centro de Apoio Científico-Tecnolóxico à Investigación (CACTI), Vigo, Spain). The images were processed using ImageJ software (version 1.53t, National Institutes of Health (NIH), Bethesda, MD, USA).

### 2.5. Photocatalytic Assays

For the photodegradation assays, a homemade irradiation setup was used, following previous works [27,29]. This system incorporates a 100 W Xenon arc lamp, a 400 nm long-pass filter, two lenses (for collimation and focusing) and a sample cuvette holder. First, all the samples were left for 30 min in the dark under stirring in order to reach the initial adsorption equilibrium of dye/nanoparticle. Then visible-light irradiation was applied, with aliquots taken throughout the assay. All the aliquots were centrifuged to remove the solid content. A dark assay was conducted under the same conditions as the light irradiation as a control to account for possible slow adsorption kinetics and to discriminate from the photocatalytic process.

UV/Vis absorption spectra were analyzed by fitting to a sum of Gaussian functions and a dispersive Rayleigh background to eliminate the influence of possible non-sedimented nanoparticles. The resulting absorption maximum was then proportional to the concentration of MG.

Three different calculations were made to evaluate the different stages of dye removal. First, the initial adsorption capacity of the samples was analyzed by Equation (1), where C_initial_ (10 mg L^−1^) is the initial concentration of dye and C_30_ is the concentration after 30 min in the dark. To assess the final removal after applying the studied conditions (dark or visible), Equation (2) was applied, where C is the dye concentration after a certain reaction time. To calculate the photodegradation rate constant, k (min^−1^), a pseudo-first order kinetics model was applied, considering, when applicable, a residual value, C_∞_, with the fraction f_∞_ = C_∞_/C_0_, as shown in Equation (3).

(1) $$\mathrm{Initial}\;\mathrm{adsorption}\;(\%)=100-\frac{\left(C_{initial}-C_{30}\right)\times 100}{C_{initial}}$$

(2) $$\mathrm{Final}\;\mathrm{removal}\;(\%)=\mathrm{Initial}\;\mathrm{adsorption}\;(\%)+(100-\frac{\left(C_{30}-C\right)\times 100}{C_{30}})$$

(3) $$C/C_{0}=f_{\infty }+{\left(1-f_{\infty }\right)e}^{-kt}$$

## 3. Results and Discussion

### 3.1. Characterization of NPs

#### 3.1.1. UV/Vis Absorption Spectra

**Figure 3:**
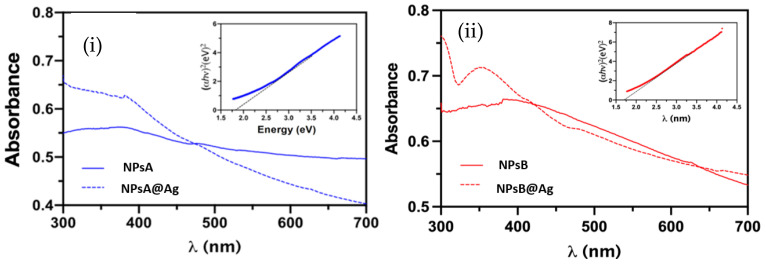
UV-Vis absorption spectra of nanoparticle solutions (1 mg mL^−1^). (**i**) NPsA and NPsA@Ag. Inset: Tauc plot for NPsA. (**ii**) NPsB and NPsB@Ag. Inset: Tauc plot for NPsB.

The UV/Vis absorption spectra of the two types of Zn_0.5_Mg_0.5_Fe_2_O_4_ nanoparticles, with and without silver deposition, were measured (Figure 3). Figure 3i shows the absorption spectra of NPsA and NpsA@Ag, while Figure 3ii displays the spectra of NPsB and NPsB@Ag. Samples containing silver show maximum absorption peaks between 300 and 400 nm [40], confirming the presence of silver at the nanoparticles’ surface. The band gap of the synthesized NPsA and NPsB samples was also calculated using a standard Tauc plot. Band gaps of 1.8 eV and 1.7 eV were calculated for NPsA and NPsB, respectively. These values, although slightly lower, are in accordance with other studies regarding zinc and magnesium ferrites, showing the capacity of light harvesting in the visible spectrum [41,42]. The absorption spectra also show that the photodeposition step originates a better dispersion in aqueous medium, especially for the NPsA@Ag sample.

#### 3.1.2. X-ray Diffraction (XRD)

**Figure 4:**
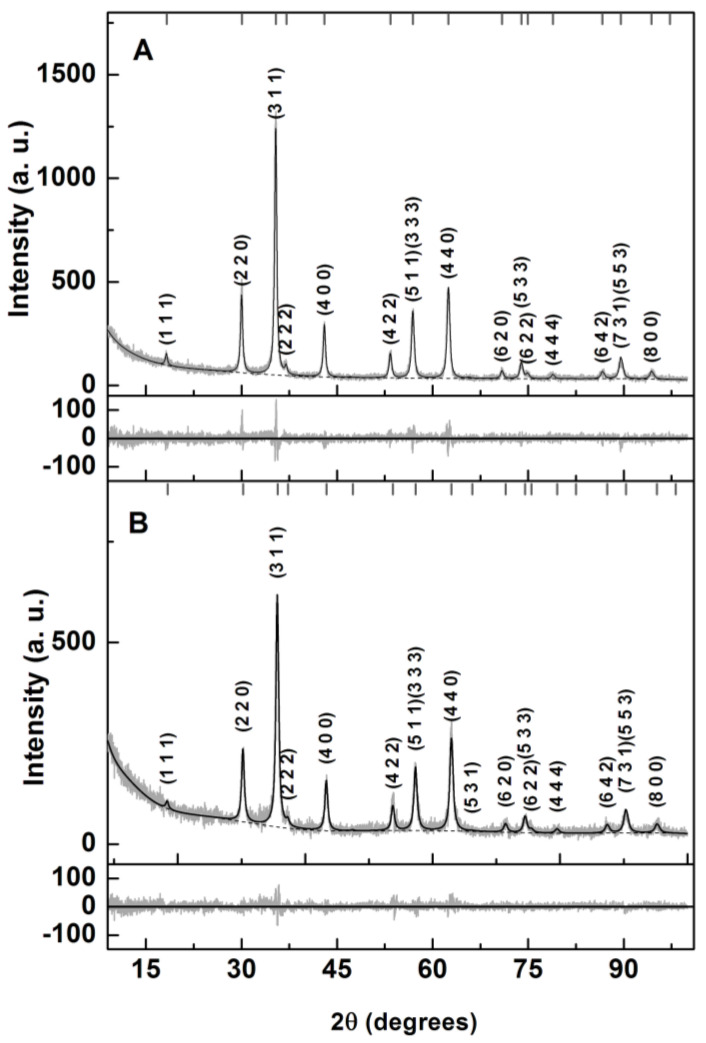
XRD diffractograms and corresponding Rietveld analyses of obtained Zn/Mg mixed ferrites by sol-gel (**A**) and solvothermal (**B**) methods.

The XRD results displayed in Figure 4 allowed us to confirm the crystallinity of the prepared NPs. Rietveld analyses of the experimental diffractograms were performed with Profex software [43] based on BGMN [44]. The zinc ferrite CIF file no. 2360015 (space group Fd-3m:1) was changed so that a stoichiometric distribution of Mg and Zn cations across the tetrahedral and octahedral sites occurred within a fully inverted spinel structure [29]. The main results are shown in Table 1. 

Reasonable fits were obtained, with R_P_ values of 9.2 and 10.8 for the NPsA and NPsB nanoparticles, respectively. The obtained lattice parameters were slightly smaller than the ones reported for Zn and Ca mixed ferrite obtained by a similar sol-gel method [29]. This was an expected result, as Mg^2+^ ions have a smaller radius than Ca^2+^. For size prediction, the implementation of the size-broadening effect in BGMN allowed size estimations of 21.5 nm for NPsA and 16.6 nm for NPsB.

#### 3.1.3. Transmission Electron Microscopy (TEM)

**Figure 5:**
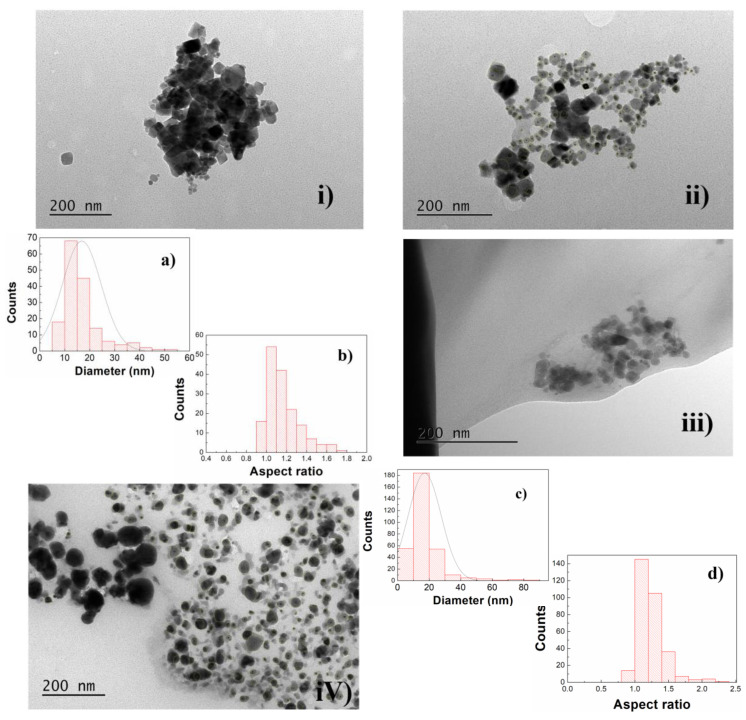
TEM images of Zn_0.5_Mg_0.5_Fe_2_O_4_ before and after silver functionalization. (**i**,**iii**) NPs synthesized by sol-gel (NPsA) and solvothermal (NPsB) methods, respectively; (**ii**,**iv**) the same NPs but with prior solvent cleaning; (**a**) size distribution of NPsAc and (**c**) size distribution of NPsBc; (**b**,**d**) aspect ratios of NPsAc and NPsBc, respectively.

TEM images of the synthesized NPs are presented in Figure 5. Both NPsA and NPsB primarily exhibited a cubic structure. Only minor differences in size and shape were observed between the nanoparticles produced by the two preparation methods.

The most relevant features were the differences observed after the intermediate cleaning process and after silver photodeposition. In the first case, a visible difference was noted between NPs without and with a cleaning step, with fewer aggregates presented in the non-cleaned NPs. For that reason, both size distributions were analyzed in these images, as the NPs appeared much larger and more individualized, allowing for better definition. For size estimation, the nanoparticles were manually outlined, and a circle with the same area was considered to estimate the diameter. The synthesized NPsAc had an average size of 14.7 ± 5.2 nm, while NPsBc had an average size of 17.4 ± 8.0 nm. These values were in accordance with the XRD results. For aspect ratio estimation, ImageJ software was used to fit a rectangle to each outlined particle. Then a histogram based on the ratio of the longer side over the smaller one was constructed (Figure 5b,d).

#### 3.1.4. Zeta-Potential pH Profiles

**Figure 6:**
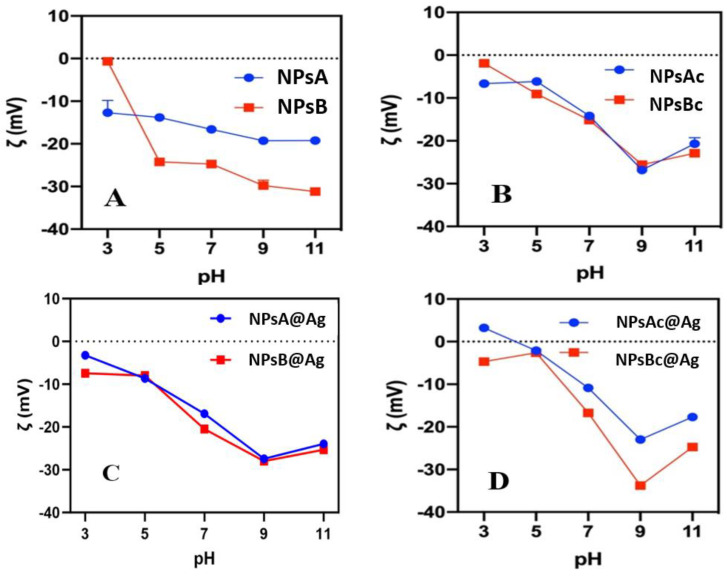
ζ-potential vs. pH profiles of Zn_0.5_Mg_0.5_Fe_2_O_4_ synthesized by different methods, post-synthesis cleaning process and functionalized with silver. (**A**) NPsA and NPsB samples without post-synthesis cleaning; (**B**) NPsA and NPsB samples cleaned with DMSO and THF; (**C**) NPsA@Ag and NPsB@Ag samples without post-synthesis cleaning; (**D**) NPsAc@Ag and NPsB@Ag samples cleaned with THF and DMSO.

The zeta(ζ)-potentials of Zn_0.5_Mg_0.5_Fe_2_O_4_ and Ag@Zn_0.5_Mg_0.5_Fe_2_O_4_ were measured at pH values from 3 to 11 (Figure 6). These measurements aimed to understand the influence of the post-synthesis cleaning processes (with organic solvents THF and DMSO) on the surface charge and the influence of silver on the cleaned nanoparticles. Overall, the nanoparticles exhibited a negative surface charge, with the magnitude tendentially decreasing at lower pH values. Ferrites typically present isoelectric points near pH = 7. The absence of positive values at lower pH levels indicates that buffer molecules such as phosphate, citrate and borate adsorb into the surface of the NPs. Nevertheless, despite being composed of the same constituents combined in the same ratio, there was a significant difference between the ζ-potential of Zn_0.5_Mg_0.5_Fe_2_O_4_ NPs synthesized by the two different methods (NPsA and NPsB). In contrast to NPsA, NPsB demonstrated a high-variation ζ-potential profile vs. the pH of the medium, ranging from nearly to 0 mV at pH 3 to −30 mV at pH 11. These differences may be attributed to the use of oleic acid in the solvothermal synthesis and its binding to the surface of the NPs, as its pK_a_ is around 5.

Interestingly, after the cleaning step, the ζ-potential pH profiles were similar for both type of NPs (Figure 6B). This indicates that both oleic acid and the molecules remaining at the particles’ surface after the sol-gel process were effectively removed by the cleaning procedure.

Silver photoreduction in the presence of methanol is expected to produce positively charged surfaces due to the adsorption of silver monocations [26]. Thus, the silver photodeposition process tends to increase the ζ-potential, turning it less negative. However, this was not observed in NPsA@Ag and NPsB@Ag samples (without cleaning), which may be due to the leaching of silver from the surface of the NPs, which is common in basic media. In the cleaned samples, the same was not observed. Better binding of silver to the NPs’ surface after the post-synthesis cleaning can explain this behavior. Also noteworthy is the positive ζ-potential in the NPsA@Ag sample, a characteristic that can be explored in the future by using negatively charged pollutants in low-pH wastewater. NPsB@Ag without any cleaning demonstrated a less negative overall ζ-potential when compared to NPsB. The cleaned NPsBc and NPsBc@Ag showed an increase in ζ-potential (less negative) from pH = 7 to acidic media.

#### 3.1.5. Magnetic Properties

The magnetic properties of Zn_0.5_Mg_05_Fe_2_O_4_ NPs are intrinsically related to the incorporation of magnesium into the ferrite structure [45]. It was previously reported [46] that the inclusion of magnesium improves the overall magnetization of the NPs while decreasing the crystalline size compared to ZnFe_2_O_4_.

**Figure 7:**
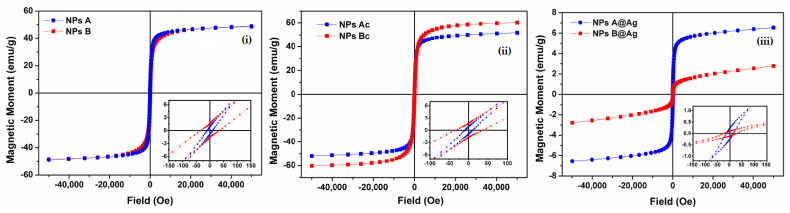
Magnetization hysteresis loops of zinc/magnesium ferrites without and with depositions of silver at 300 K. (**i**) NPsA and NPsB; (**ii**) NPsAc and NPsBc; (**iii**) NPsA@Ag and NPsB@Ag. Insets: enlargement of the loops in the low field region.

Furthermore, Zn^2+^ and Mg^2+^ are divalent cations that prefer tetrahedral positions (A) in the ferrite structure, reducing the position exchanges between cations in tetrahedral and octahedral sites, with improvement of the maximum magnetization [47]. The magnetic hysteresis loops of the samples were investigated by the SQUID technique to understand the influence of synthesis methods, post-synthesis cleaning and silver photodeposition. The results are shown in Figure 7 and Table 2.

NPsA and NPsB did not show a significant change in their magnetic profiles. Saturation magnetizations (M_s_) of circa 49 emu g^−^^1^ for NPsA and 48.5 emu g^−^^1^ for NPsB were obtained (Figure 7i). Both NPs exhibited superparamagnetic behavior, with a ratio of M_r_/M_s_ below 0.1, meaning that more than 90% of the magnetization was lost when no magnetic field was applied. NPsAc demonstrated a slight increase in M_s_ due to the cleaning process, increasing to 51.8 emu g^−^^1^ (Figure 7ii). However, the largest difference was observed in the NPsBc sample, increasing to 60.2 emu g^−^^1^ (Figure 7iii), indicating that there were still some reaction products in these samples and that the cleaning process using organic solvents (THF and DMSO) contributed effectively to their removal. The M_s_ value for NPsBc was higher than that previously observed for mixed calcium/magnesium ferrites obtained by the same method [39].

On the other hand, the photodeposition of silver substantially reduced the M_s_ values of the NPs to 6.57 emu g^−^^1^ and 2.79 emu g^−^^1^ for NPsA@Ag and NPsB@Ag, respectively. This reduction is attributed to the contribution of the non-magnetic Ag coating, evidencing successful deposition of silver on nanoparticles’ surfaces.

### 3.2. Photocatalytic Assays

To assess the photocatalytic activity of the synthesized NPs, different assays were performed using a model dye solution with MG. Table 3 summarizes all the results of the photocatalytic assays performed, and their discussion is detailed in the following sections.

#### 3.2.1. Adsorption Phenomena on NPs

**Figure 8:**
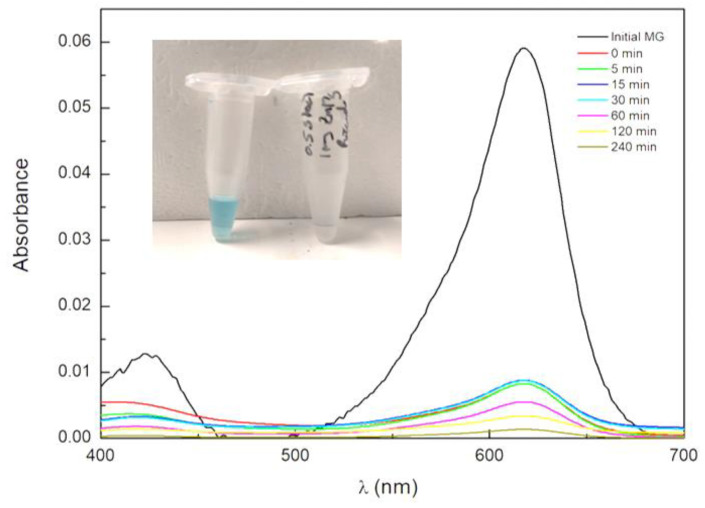
Absorption spectrum of MG at initial concentration of 10 mg L^−1^, and adsorption spectrum over photocatalytic reaction time for NPsA as an example. Inset: photographs at initial and final incubation times. The first 30 min correspond to dark conditions.

As previously mentioned, ferrite nanoparticles present low photocatalytic activity due to the high recombination of electrons/holes. However, despite the expected inefficient photocatalytic activity, high removal of MG was observed (Figure 8). During the first 30 min assay under dark conditions, a large percentage of dye was removed from the aqueous medium. This is explained by the negative surface charge of the zinc/magnesium ferrites, as demonstrated in ζ-potential pH studies (Figure 6), which favored the affinity between the negatively charged NPs and the positively charged dye [48]. The NPsA showed the highest adsorption capacity, achieving an average of 86% removal in just 30 min under dark conditions, while NPsB demonstrated a significantly lower adsorption capacity, with an average adsorption of 48%. These differences in adsorption for the two samples may be due to the interference of residues from the synthesis process.

**Figure 9:**
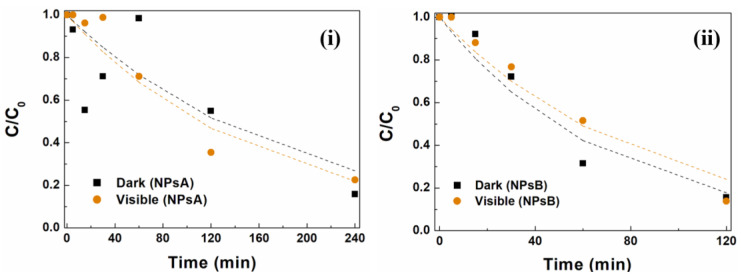
MG degradation curves using zinc/magnesium ferrites: (**i**) NPsA under dark and visible-light conditions over 240 min; (**ii**) NPsB under dark and visible-light conditions over 120 min.

In order to find out whether adsorption was the main process involved in MG removal, prolonged assays were conducted under both dark and visible-light conditions. The results demonstrated similar behavior between the two conditions, with increases in dye removal of 97% for NPsA and 92% for NPsB (Figure 9). The similarity between dark and visible-light conditions and the highly efficient removal of MG indicate that the presence of light is not decisive for dye removal.

#### 3.2.2. Influence of Silver on NP Catalytic Activity

The increase in ζ-potential with the deposition of silver was expected to decrease the affinity between the NPs and the dye. This was inferred from the initial adsorption results, which are indicated in Table 3. To evaluate the influence of silver deposition, different assays were performed under both dark and visible-light conditions. First, photolysis (pollutant decomposition by direct action of light) was tested, demonstrating an almost negligible degradation of 8.7% after 240 min, with a degradation rate of 0.0004 min^−^^1^.

**Figure 10:**
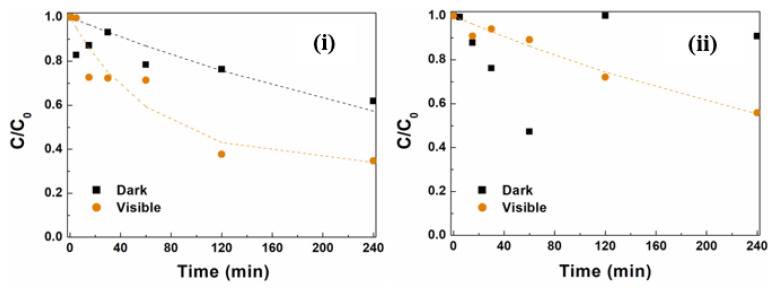
MG degradation curves using zinc/magnesium ferrites functionalized with silver, under dark and visible-light conditions over 240 min. (**i**) NPsA@Ag and (**ii**) NPsB@Ag.

Figure 10 shows the degradation curves for the NPsA@Ag and NPsB@Ag samples under dark and visible-light conditions. For NPsA@Ag, a saturation effect of the photodegradation over time was observed. This was accounted for by considering the non-decaying fraction of MG molecules, f_∞_ (Equation (3)). Under visible light, NPsA@Ag showed significantly higher degradation, with nearly twice the removal activity and an overall removal increase of 22% at a rate constant of 0.0153 min^−^^1^. Under dark conditions, NPsB@Ag showed up-and-down behavior, possibly due to adsorption/desorption phenomena occurring at the surface. Under visible-light irradiation, removal activity of 44% at a rate of 0.0025 min^−^^1^ was observed, which was much lower than that obtained with NPsA@Ag. The results observed with both NPs demonstrated the influence of silver on photocatalytic activity and pointed to a higher number of active sites available at the surface of NPsA@Ag when compared with NPsB@Ag.

#### 3.2.3. Influence of NP Concentration

**Figure 11:**
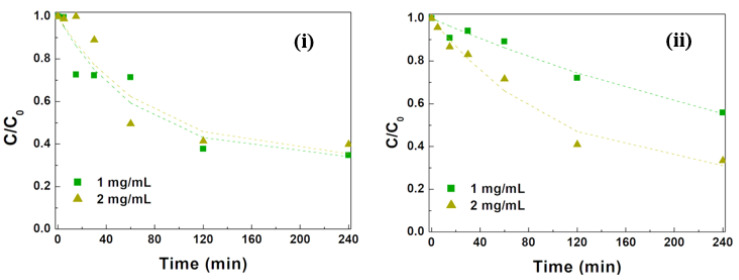
MG degradation curves using two concentrations of zinc/magnesium ferrite nanoparticles functionalized with silver: (**i**) NPsA@Ag and (**ii**) NPsB@Ag.

The use of different concentrations of NPs was expected to affect the photocatalytic degradation of pollutants. Therefore, assays with varying NPs concentrations were performed to analyze the influence of this parameter on the degradation of MG. A decrease in initial adsorption occurred with increasing NP loads, although the available surface sites were expected to increase. One possible explanation is aggregation of the NPs, originating an effective decrease in the exposed surface area. Another factor contributing to the adsorbed amount being less than expected was the comparable concentrations of adsorbate (C_A_) and NP surface adsorption sites (C_S_). The concentration of MG that was used was 27.4 µM. Considering a spherical NP of 20 nm in size, with a density of 4.94 g/cm^3^ (calculated from XRD analysis), and that each MG molecule occupies a circle with a 10 Å radius of the surface area, the adsorption surface site concentration can be estimated as 32.1 µM in the case of a 1 mg mL^−^^1^ NP load. Langmuir isotherm is not valid in these conditions and, considering a simple adsorption equilibrium, the following equation (Equation (4)) can be obtained:

(4) $$K=\frac{\theta }{\left(C_{A}-\theta C_{S}\right)\left(1-\theta \right)}\Leftrightarrow \theta^{2}-\left(\frac{C_{A}}{C_{S}}+1+\frac{1}{KC_{S}}\right)+\frac{C_{A}}{C_{S}}=0$$

where K is the adsorption equilibrium constant, and θ is the fraction of surface sites that are occupied by adsorbed molecules. If the adsorption constant is 5 × 10^4^ M^−^^1^, a value of 0.414 is obtained for θ, corresponding to a concentration of 13.3 µM of MG adsorbed. If the particle concentration is doubled to 2 mg mL^−^^1^, the corresponding values are 0.296 and 19.0 µM. Thus, the adsorbed quantity does not double but instead only increases by a factor of 1.43. Thus, the observed decrease in initial adsorption indicates the presence of aggregation phenomena that increase with the NP load. This aggregation can be promoted by the neutralization of negative charges on the NP surfaces by positively charged MG molecules, which act as a bridge. This process also leads to solvent entrapment and, consequently, to localization of MG molecules within the aggregate. The MG molecules are expected to be much less efficiently photodegradated and thus can be the origin of the fraction, f_∞_, of MG molecules that do not become degradated during the studied timescale. The degradation curves of NPsA@Ag and NPsB@Ag at different concentrations (1 and 2 mg mL^−^^1^) are shown in Figure 11.

In the case of NPsA@Ag, the photodegradation results were very similar with both concentrations, indicating that the rise in NP concentration did not significantly increase the number of surface photoactive sites, with the overall removal efficiency being higher with 1 mg mL^−^^1^ due to higher initial adsorption and a slightly faster photodegradation process. Another factor that may have played a role is a higher attenuation of irradiation light intensity with increasing NP concentrations on account of larger sample absorbance. In fact, an increase in absorbance from 0.5 to 1.0 made the transmitted light intensity fraction (at 1 cm depth) decrease from 0.61 to 0.37. This slowed the photodegradation rate, as NP photoexcitation efficiency decreased.

For the case of NPsB@Ag, the photodegradation rate increased 2.3 times when the NPs concentration was doubled. This indicates that the aggregation effect was less pronounced for NPsB@Ag, leading to an effective increment of active surface sites with an increase in the NP load. Nevertheless, despite this increased efficiency, it only matched results the obtained with 1 mg mL^−^^1^ of NPsA@Ag (Figure 11).

#### 3.2.4. Influence of Ag Photodeposition Time

**Figure 12:**
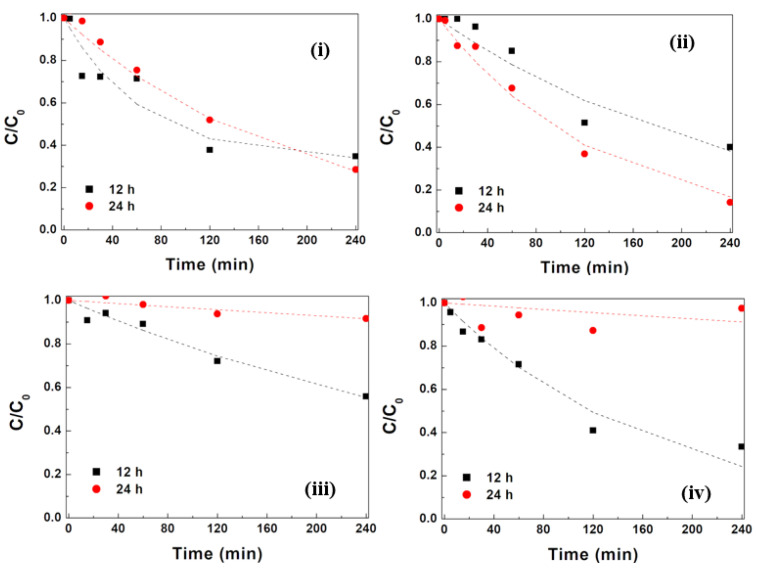
MG degradation curves using zinc/magnesium ferrite nanoparticles functionalized with silver after 12 h and 24 h of silver deposition time: (**i**,**ii**) NPsA@Ag at concentrations of 1 mg mL^−1^ and 2 mg mL^−1^, respectively; (**iii**,**iv**) NPsB@Ag with concentrations of 1 mg mL^−1^ and 2 mg mL^−1^, respectively.

In order to study the influence of the silver photodeposition time on the photocatalytic activity of the mixed ferrites, studies of MG photocatalytic removal were carried out with NPs with 12 h and 24 h of silver deposition time and for two NP concentrations (1 mg mL^−^^1^ and 2 mg mL^−^^1^). The results for all tested samples are exhibited in Figure 12.

NPsA@Ag showed a decrease in initial adsorption, which was attributed to the larger amount of deposited silver on the surface, making it much less negative. A slight increase in initial adsorption with the doubling of the NP concentration points to a much lower presence of NP aggregation. This was confirmed by the absence of a non-photodegradable population of MG molecules (f_∞_ = 0) and by a slight increase in the photodegradation rate constant with the NP concentration. Overall, better removal efficiencies of 84% and 88% were obtained for NPsA@Ag at concentrations of 1 mg mL^−^^1^ and 2 mg mL^−^^1^, respectively.

For NPsB@Ag, a distinct behavior was observed, i.e., an increase in Ag photodeposition time resulted in high suppression of initial adsorption and almost negligible photocatalytic activity. This may be due to the nearly complete overcoating of the exposed mixed ferrites’ surfaces with silver, leading to very low MG adsorption onto the NP surfaces and negligible efficiency in the conversion of water molecules into hydroxyl or superoxide radicals, which are normally involved in the photodegradation mechanism [29].

#### 3.2.5. Influence of NP Surface Cleaning

**Figure 13:**
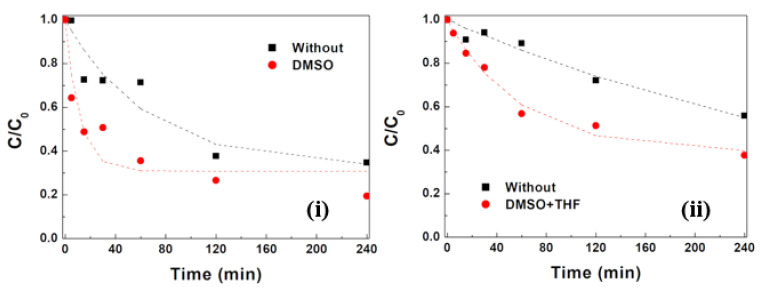
Degradation curves of MG (initial concentration of 10 mg L^−1^) with (**i**) NPsAc@Ag without and with a cleaning step with DMSO; (**ii**) NPsBc@Ag without and with a cleaning step with DMSO + THF.

Surface cleaning is essential if, due to the presence of residues of the synthesis process, a significant part of NPs’ surfaces is inaccessible to further functionalization and to the photogeneration of the reactive radicals that normally initiate the photodegradation process. These unwanted residues especially occur in synthesis by the solvothermal method, and their removal can be facilitated by the use of organic solvents. To test the impact of these molecules on silver surface photodeposition and in the photocatalytic activity of the resulting NPs, a cleaning process with DMSO or DMSO and THF was used. The results of the NPs cleaning on the photocatalytic activity towards MG are shown in Figure 13.

The impact of this step was particularly notable in NPsBc@Ag, which were synthesized by the solvothermal method, for which an increase in the overall adsorption was observed. Furthermore, during the light exposure, enhanced photoactivity was observed (Figure 13ii), which was attributed to a larger surface availability, and an 87.6% MG removal efficiency was reached at a rate constant of 0.017 min^−^^1^. This positive influence was also observed in NPsAc@Ag sample, indicating that even in the sol-gel process, some synthesis residues remain attached to the nanoparticle surfaces. The most favorable overall outcome was observed in this assay, where a final removal of 95.0% and a degradation rate of 0.091 min^−^^1^ were achieved. Based on these results, it is clear that this intermediate cleaning step seems to be crucial for the optimization of the photodegradation efficiency of zinc/magnesium ferrite NPs with photodeposited silver. The latter rate constant is ca. five times higher than the ones previously reported for MG degradation with cobalt oxide NPs (modified with citric acid and oleic acid) synthesized by sol-gel [49] and with copper oxide and copper cobaltite photocatalysts [50], as well as using SmMnO_3_-ZnO [51] (Table 4).

Also, the reaction rate with NPsAc@Ag was four times faster than that achieved with rGO-Fe_3_O_4_/TiO_2_ [53] and about twice as effective as ZnO [52] despite the photocatalyst/MG ratio being two to three times higher. Moreover, some of the works used simulated sunlight, which has a UV content much higher than the visible light employed in this study (7% [56] vs. 0.2%), although the visible-light irradiance of the optical system used was slightly higher (~600 W/m^2^ [56] vs. 650 W/m^2^).

Considering the use of visible light and the rate constant for MG degradation, together with the percentage of dye removal, we can conclude that NPsAc@Ag nanoparticles are particularly advantageous for MG removal from industrial effluents. Moreover, a real possibility for magnetic recovery and reuse of these nanoparticles (or retention within the reactor for multiple treatment cycles) is expected, as previously observed for zinc/calcium ferrites with photodeposited silver [29]. This is favored, as the neat zinc/magnesium ferrites exhibit improved magnetic properties compared to zinc/calcium ferrites [27], with a higher saturation magnetization. To the best of our knowledge, this work is the first study employing silver-functionalized zinc/magnesium ferrite nanoparticles in the photodegradation of an industrial dye.

## 4. Conclusions

Zinc/magnesium mixed ferrite nanoparticles were synthesized both by sol-gel and solvothermal methods and further functionalized with silver. The synthesized mixed ferrite nanoparticles generally exhibited a cubic shape, a crystalline structure and superparamagnetic behavior. The ability of the silver-functionalized nanoparticles for photocatalysis using visible light was demonstrated, with an almost complete removal of MG dye for the optimized nanoparticles. Zn_0.5_Mg_0.5_Fe_2_O_4_ NPs act as photocatalysts by generating reactive species that initiate the MG degradation process upon light absorption. The presence of metallic silver reduces the electron/hole recombination rate, enhancing the photocatalytic activity.

It was demonstrated that applying an intermediate cleaning step using DMSO or DMSO + THF before the silver photodeposition, resulted in a significant improvement in photocatalytic properties. The adsorption contribution for MG removal was ca. 89% for nanoparticles prepared by sol-gel and 83% for the ones prepared using the solvothermal method.

The prepared silver-functionalized mixed ferrites had suitable properties for future industrial application, taking advantage of the possibility for photocatalyst magnetic recovery and reuse.

## Figures and Tables

**Table 1:** Selected parameters from Rietveld analysis using BGMN.

Sample	O_x,y,z_ (*)	*i* (**)	Phase Size (nm) Lattice Constant (nm) Zn, Mg Ferrite	R_P_	χ2
NPsA	0.3783	1 (^+^)	21.5 0.8401	9.2	1.3
NPsB	0.3760	1 (^+^)	16.6 0.8344	10.8	1.5

(*) Value of O_x,y,z_ in CIF file 2300615 is 0.2535. (**) inversion degree. (^+^) fixed value.

**Table 2:** Saturation magnetization (M_s),_ remanent magnetization (M_r_), coercive field (H_c_) and ratio M_r_/M_s_.

NPs Samples	M_s_ (emu g^−1^)	M_r_ (emu g^−1^)	H_c_ (Oe)	M_r_/M_s_
NPsA	48.98	0.91	9.57	0.02
NPsB	48.53	1.61	33.94	0.03
NPsAc	51.82	0.91	10.26	0.02
NPsBc	60.17	1.63	36.18	0.03
NPsA@Ag	6.57	0.24	15.02	0.04
NPsB@Ag	2.79	0.11	26.65	0.04

**Table 3:** Results obtained by the different assays performed with Zn_0.5_Mg_05_Fe_2_O_4_ NPs with and without silver functionalization.

Sample	Synthesis	Light Condition	[NPs] (mg mL^−1^)	Initial Adsorption (30 min Dark) (%)	Average Initial Adsorption	Removal Activity under Dark/Visible Conditions (%)	Time (min)	Overall Removal Efficiency (%)	Rate Constant (min^−1^)|f_∞_
**Photolysis**	-	Visible	-	n.a.	n.a.	8.7	240	8.7	0.0004
**NPsA**	Sol-gel method	Dark	1	86.7	86.5	84.2	240	97.9	n.a.
		Visible		86.2		77.4		96.9	0.0063
**NPsB**	Solvothermal method	Dark	1	50.8	48.2	84.4	120	92.3	n.a.
		Visible		45.6		86.2		92.5	0.0118
**NPsA@Ag**	Sol-gel method, 12 h Ag photodeposition	Dark	1	40.7	49.2	38.2	240	63.3	n.a.
		Visible	1	57.8		65.4		85.5	0.0153|0.3230
		Visible	2	48.5	n.a.	60.1		79.5	0.0138|0.3297
	Sol-gel method, 24 h Ag photodeposition	Visible	1	40.5	n.a.	73.0		83.9	0.0053
		Visible	2	41.7	n.a.	80.0		88.3	0.0057
	Sol-gel method, cleaned, 12 h Ag photodeposition	Dark	1	86.0	80.2	22.7		89.2	n.a.
		Visible	1	84.4		80.5		95.0	0.0910 |0.3070
**NPsB@Ag**	Solvothermal method, 12 h Ag photodeposition	Dark	1	24.7	36.3	n.a.	240	n.a.	n.a.
		Visible	1	47.9		44.1		71.0	0.0025
		Visible	2	39.8	n.a.	66.7		79.9	0.0098|0.2410
	Solvothermal method, 24 h Ag photodeposition	Visible	1	6.1	n.a.	8.4		14.0	0.0004
		Visible	2	6.4	n.a.	2.5		8.7	0.0004
	Solvothermal method, cleaning step, 12 h Ag photodeposition	Dark	1	77.4	n.a.	24.2		82.8	n.a.
		Visible	1	67.3	n.a.	62.2		87.6	0.0170|0.3880

**Table 4:** Comparison of different nanostructures used for degradation of MG under simulated sunlight or visible light.

Nanomaterial	MG Concentration (mg/L)	Concentration Photocatalyst (mg/mL)	Light Source	Degradation (%)	Time (min)	Rate (min^−1^)	Reference
**ZnO/In_2_Cu_2_O_5_**	2.19	0.17	Visible light	93.9	120	0.0226	[37]
**YMnO_3_-doped TiO_2_**	2.19	0.2	Visible light	95.4	120	0.0228	[38]
**Citric acid-capped cobalt oxide**	3.65	0.5	Simulated sunlight	91.2	100	0.0128	[49]
**SmMnO_3_-ZnO**	2.19	0.12	Visible light	91.7	120	0.0190	[51]
**ZnO (ZEDTA)**	3.65	0.2	Simulated sunlight	94.1	41	0.0582	[52]
**rGO-Fe_3_O_4_/TiO_2_**	5.5	0.15	Visible light	99	55	0.0224	[53]
**CH/ZnO**	5	0.5	Visible light	100	90	-	[54]
**CH/Ce-ZnO**	5	0.3	Visible light	100	60	-	[55]
**NPsAc@Ag**	10	1	Visible light	95	240	0.0910	This work
**NPsBc@Ag**	10	1	Visible light	87.6	240	0.0170	This work

## Data Availability

The original contributions presented in the study are included in the article, further inquiries can be directed to the corresponding author.
